# Perceived versus Observed Patient Safety Measures in a Critical Care Unit from a Teaching Hospital in Southern Colombia

**DOI:** 10.1155/2016/2175436

**Published:** 2016-02-18

**Authors:** Jorge Hernan Montenegro, Adriana Fernanda Romero, Paola Andrea Tejada, Sandra Ximena Olaya, Andres Mariano Rubiano

**Affiliations:** ^1^South Colombian University, Neiva, Colombia; ^2^Neiva University Hospital, Neiva, Colombia; ^3^Trauma and Emergency Service, Neiva University Hospital, Neiva, Colombia; ^4^MEDITECH Foundation, Neiva, Colombia

## Abstract

*Introduction*. Patient safety is an important topic. The purpose of this study is to evaluate the perceived versus observed patient safety measures (PSM) in critically ill patients in a teaching hospital in Latin America.* Materials and Methods*. The level of perceived patient safety was evaluated with the patient safety hospital survey. Three months later, a qualitative study was conducted, including video recording of procedures, graded according to adherence to PSM. Levels of adherence were scored during patient mobilization (PM), placement of central catheters (PCC), other invasive procedures (OIP), infection control (IC), and endotracheal intubation (ETI).* Results*. The perceived adherence of PSM in the prestudy survey was considered fair by 89.1% of the ICU staff. After the survey, 829 ICU procedures were video-recorded. Mean observed adherence for fair patient safety measures was 20.8%. Perceived adherence was higher than the real patient safety protocol measures observed in the videos.* Conclusion*. Perception of PSM was higher than observed in the management of critically ill patients in a teaching hospital in southern Colombia.

## 1. Introduction

Patient safety has been defined as the reduction or mitigation of unsafe acts within the health services [1]. Unsafe acts are not intentional and are associated with errors in the application of standardized protocols of care. These unsafe acts affect the outcome of patients and may vary between 1 and 22% depending on the service where they are measured and the type of institution and the method used for the measurement [2]. It has been reported that between 30 and 70% of them are preventable and can have a significant impact in terms of cost of care and patient outcome, including aspects like prolongation of the hospital stay, residual disability, and mortality.

Due to this, in the last few years the agenda of main national and international agencies that promote patient safety seeks to establish guidelines and protocols in order to minimize the possibility of unsafe acts during the care of any patient, with emphasis on the most critical ones [3]. The scientific evidence documented in the past few years related to unsafe acts in health care has forced decision-makers to establish policies and guidelines related to the prevention on these acts. In general, these policies and guidelines are based on three main topics: access, efficiency, and quality. Quality includes the detection of unsafe acts and its correction in order to improve patient safety. Unsafe acts include a range of acts like inappropriate hand washing (sometimes unperceived acts) to more serious acts, like inappropriate use of protocols for invasive vascular or airway procedures, which may result in injury, disability, or death [4].

**Figure 1:**
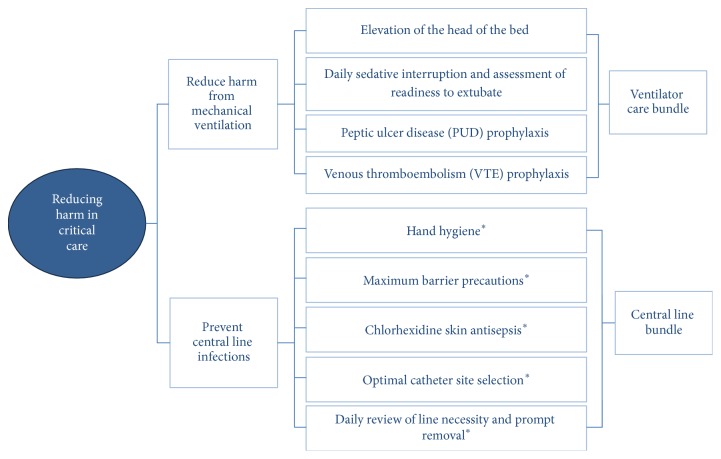
Example of ICU bundle of cares interventions. This study has been focused on aspects related to infection control similar to the central line bundle interventions (*∗*). Adapted from [8].

Intensive care and patient safety experts have developed intense campaigns in order to minimize harm on the critical care patients. Aspects related to human factors and their interaction with devices, technology, and medications have been analyzed and organized in bundles of care with the aim of minimizing unsafe acts (Figure 1) [5–8]. One of the most frequent errors is associated with infection control followed by failures in monitoring during the intrahospital transfers and failures in adherence to protocols during invasive procedures. These unsafe acts represent subsequently associated complications such as infections and episodes of hypoxia or hypotension.

Data from studies supported by the World Health Organization show that cost of intrahospital infections exceeds 29 billion dollars annually and also that one in every 135 hospitalized patients has an infection acquired in the hospital. The vast majority of these infections are preventable if appropriate adherence with the bundles of care is maintained [9].

In low- and middle-income countries, studies regarding patient safety in ICUs are very limited. The few studies on this topic almost always suggest a proposal for further research [1, 3, 10, 11]. The three main axes for future studies include the following [12–14]:

1. Identification of the climate of patient care quality in different intrahospital scenarios.
2. Determinations of what are the possible failures during the care of the patient.
3. Creation of checklists in different scenarios during critical moments of the care: including resuscitation of the patient and monitoring during the bath or patient transfer and during invasive procedures.

Our objective with this study was to compare perceived patient safety measures compared with observed measures in the management of critical care patients in a university hospital in southern Colombia. This issue is important for evaluation as false perception of safety in environments of real unsafe acts brings an important bias over the quality of care in patients, affecting future decisions on patient safety policies.

## 2. Materials and Methods

We perform an exploratory descriptive study crossed with direct observations. A survey including aspects of perceived adherence to patient safety measures was performed 3 months before the start of the study using the “Hospital Survey on Patient Safety” questionnaire [15]. Posteriorly, a qualitative analysis was conducted between July and September of 2012, with video cameras in 6 randomized cubicles of 28 from two intensive care units (ICUs) from the hospital.

An instrument based on the measures described in local patient safety protocols for the management of the critically ill patients was designed with a scale to evaluate real patient safety measures adherence in specific procedures like patient mobilization (PM), placement of central catheters (PCC), and other invasive procedures (OIP) such as thoracotomies, pleural puncture, and lumbar and abdominal punctures. Infection control (IC) and endotracheal intubation (ETI) procedure measures were also evaluated. A six-item scale was developed, including the accomplishment of basic steps required to follow adherence to each specific patient safety protocol (see supplementary file in Supplementary Material available online at http://dx.doi.org/10.1155/2016/2175436). Each item was graded with one point and the final adherence level was measured as follows:

- 0 positive items: failing patient safety measures adherence.
- 1-2 positive items: poor patient safety measures adherence.
- 3 positive items: acceptable patient safety measures adherence.
- 4-5 positive items: very good patient safety measures adherence.
- 6 positive items: excellent patient safety measures adherence.

This design was oriented to match the overall grade of Section  E of the “Hospital Survey on Patient Safety,” where the evaluated personnel was able to give a general perception over the patient safety conditions in their work unit (Appendix  1) three months before the study. This survey shows a fair perception of safety in 89.1% of the staff (excellent: 27%; very good: 37.8%; acceptable: 24.3%).

Three months after the survey, the staff of the ICU was informed of the objectives of the new study and we obtain informed consent for video recording by the local IRB. Patients or family members were informed of the 3 months of planned observations, taking all the precautions to protect patient identity. Six video cameras that start recording only with motion were distributed in 6 randomized cubicles and were focused in the main hallways with the idea of obtaining additional information over the main hand washing areas. Two independently trained members of the research team conducted the observational data collection. Some good practice cases were performed with each data collector in order to evaluate quality of the process, but interobserver variability was not evaluated. The methodology and data collection tools were piloted in a prestudy test. In each unit, the data collectors observed physicians, nurses, or technicians and answer the questions of the evaluated protocol (PM, PCC, OIP, IC, and ETI). Each procedure performed by the staff involved in the management of the critically ill patients at the ICU was recorded. However, only the abovementioned procedures were scored and other recordings were eliminated.

**Figure 2:**
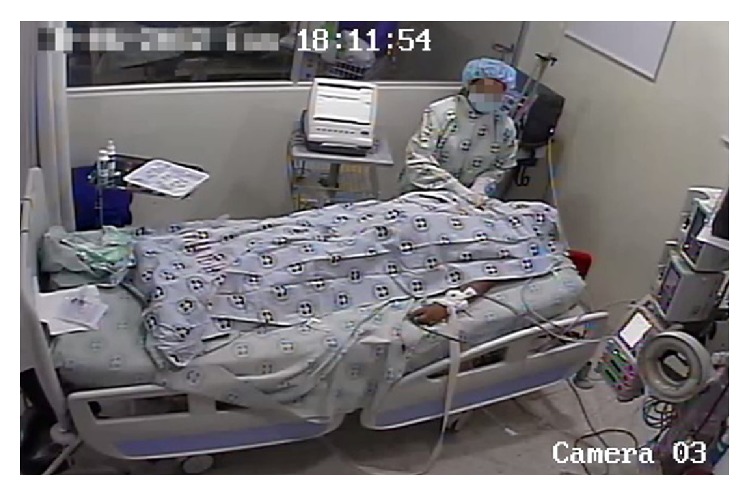
Video image of an ICU procedure while being performed by a resident of a surgical specialty in critical care patient. Photo: authors.

**Figure 3:**
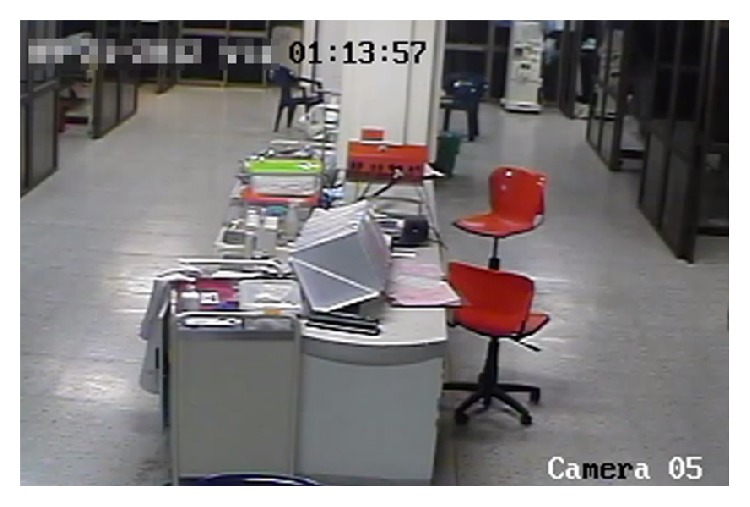
Video of area between ICU cubicles. This area was under observation for evaluating the team behavior during mobilization of patients outside the cubicles. Photo: authors.

After obtaining the videos, the instrument was administered to all procedures in the collected videos (Figures 2 and 3). Subsequent verification was conducted with personnel trained in the use of patient safety bundle checklists.

### 2.1. Statistical Analysis

A scale was developed to score the level of measure adherence for each protocol depending on the number of points answered positively in the questionnaire: 0 positive answers (failing patient safety measures adherence), 1 to 2 positive answers (poor patient safety measures adherence), 3 positive answers (acceptable patient safety measures adherence), 4 to 5 positive answers (very good patient safety measures adherence), and 6 positive answers (excellent patient safety measures adherence.). The adherence on each instrument for specific procedures was analyzed as a categorical variable (failing, poor, acceptable, very good, and excellent) using descriptive statistics of frequency. The statistical package for social sciences (SPSS) version 19.0 (Chicago, IL, USA) was used to analyze the data of the study.

### 2.2. Ethical Considerations

The local institutional ethics committee approved the study. The ethical standards of research were followed in the basis of the Declaration of Helsinki, including the ethical national guidelines for ethics in research. All participants were informed about the study and it was clarified that participation was voluntary and that they could be withdrawn at any time. There was no personal information to identify patients or health care personnel and the principal investigators, as members of the ICU staff, did not participate in the study to avoid conflict of interests or bias.

## 3. Results

The prestudy survey was performed 3 months before the start of the study. It includes 10 sections of questions of the Safety Attitude Questionnaire of the Agency for Health Research and Quality of USA. The main idea was to compare and understand the general perceived climate of patient safety in the ICU. Thirty-seven members of the staff answer the survey, including Section  6 (patient safety grade). Ten (27%) of the staff perceived an excellent patient safety environment at the ICU, 14 (37.9%) a very good one, 9 (24.3%) an acceptable level, 2 (5.4%) a poor level, and 2 (5.4%) a failing patient safety environment.

During the 7,200 hours of filming, 829 procedures were detected for evaluation of specific patient safety protocols adherence on different procedures, using the scale described in Section 2. IC (689 procedures), PM (61 procedures), OIP (52 procedures), PCC (23 procedures), and ETI (4 procedures) were analyzed and evaluated. Other procedures not relevant to the study (monitoring of vital signs, etc.) were not included. There were 511 (61.6%) procedures during weekdays, 186 (22.5%) on weekends 7a.m.–5p.m., and 132 (15.9%) during evenings (17:00–06:59). Mean level of observed patient safety measures adherence including the 5 different groups of procedures was obtained and compared with the general perception of patient safety described in the survey (Table 1).

In the protocol for IC, failing patient safety measures were present in 138 (20%) procedures and poor in 464 (67.3%); 43 (6.2%) were acceptable, 40 (5.8%) of the procedures were very good, and 4 (0.6%) were excellent. The use of sterile gloves by all the members of the team during care was not met in 93.3% of the cases (Table 2).

During PM measures of patient safety were failing in 12 (19.7%) of the cases, were poor in 19 (31.1%), were acceptable in 7 (11.5%), were very good in 18 (29.5%), and were excellent in only 5 (8.2%) of the cases. As an example in this specific protocol, during transport of patients outside the ICU, in only 17 (27.9%) cases, a specific member of the team was fully dedicated to management and monitoring of the airway. In the other 44 (72.1%) cases, the person in charge of the airway was also in charge of additional tasks related to mobilization of the stretcher or mobilization of the carrier for the portable oxygen cylinder.

Protocol measures for OIP were failing in 3 (5.8%) of the observations and were poor in 10 (19.2%), were acceptable in 16 (30.8%), were very good in 21 (40.4%), and were excellent in only 2 (3.8%) of the procedures. As an example, the use of sterile fields for invasive punctures or incisions was met only in 17.3% of the observations. The same percentage was found for the use of sterile gown, facemasks, and lenses for members of the involved team.

For the PCC protocol, poor measures were present in 7 (30.4%) cases, were acceptable in 12 (52.2%), were very good in 2 (8.7%), and were excellent in 2 (8.7%). As an example the use of sterile grown, facemasks, and lenses by all the members of the team during the process was met in only 2 (8.6%) of the procedures.

Only 2 (50%) of the ETI had very good measures following the protocol, while the remaining 2 (25%) were acceptable and poor, respectively.

## 4. Discussion

Patient safety must be a fundamental element during critical care of patients worldwide. Since the important report “To Err Is Human” health institutions have developed policies and protocols oriented to the safety of the patient. Different factors, and especially the human factor and its interaction with other humans, technology, devices, and medications, can make the everyday practice for other health care providers and the patients more difficult, resulting in unsafe acts like medical errors, adverse events, and mostly preventable deaths [13]. Processes of care in the ICU are becoming more and more complex and require commitment, continuous monitoring, and reporting of deficiencies to avoid errors in patient care [15, 16]. The report of these errors has been studied and very often health care providers do not report these events [17–21]. High adherence to protocols has been shown to improve patient safety and minimize unsafe acts in the ICU [22, 23]. Frequently, these errors are related to the administration of medications, but this event was not included in this study because it has been commonly studied and we were more centered in the already established protocols of procedures not associated with medication administration.

### 4.1. Placement of Central Catheters

Compliance to protocols and checklists for the correct placement of the central catheters would prevent associated devices infection (ADI) [24]. This aspect is a surrogate marker of patient safety and can influence morbidity, mortality, hospital stay, and additional costs [25, 26]. Anywhere from 8.2 to 38.5% of ADI result in bacteremia associated with a central line [27, 28]. Team members can improve the failing and poor patient safety measures (30.4%) observed in this study through the promotion of correct use of sterile gloves during the insertion procedures.

### 4.2. Procedures of Mobilization

Although many procedures are performed at the patient's bedside, there are others that require intrahospital mobilization like computed tomography or magnetic resonance procedures. Several studies have documented complications during the transport of patients from the ICU to perform various types of procedures, with rates from 32.4% to 75% in specific cases [29, 30]. Described complications include agitation, hypotension, and hypoxemia. In some cases unsafe acts during transport have been related to the death or severe morbidity of the patient. Adherence to protocols for transport of a critically ill patient is essential to decrease these complications. Our study shows failing and poor patient safety measures in 37.6% of cases and the most frequent unsafe act was the unavailability of a team member to be fully dedicated to airway surveillance. This failure in patient safety during transport has been reported in the literature as a common unsafe act [31]. Measures can be improved with the organization of a group for transport, with specific roles and appropriate equipment during the process.

### 4.3. Other Invasive Procedures (Thoracostomy, Lumbar Puncture, Thoracentesis, or Paracentesis)

Although invasive procedures in the critically ill patient are a common activity in ICUs, there are no studies evaluating the impact of adherence to protocols of these procedures in patient safety. However, specialized literature is clear in recommending the need for a standardized protocol for these types of procedures. The staff needs to incorporate high quality training, and if technology, such as the real time ultrasound, is incorporated, it can reduce complications and avoid unsafe acts [32–35].

### 4.4. Infection Control

The appropriate measures recommended for the management of infected or colonized patients in the ICU are important to reduce the risk of transmission to other patients or members of the care team. Infection control has been an important pillar in the development of bundles of care in the ICU environment [36–38]. Despite this very well known recommendation, our study shows that, in 87.3% of the cases, the care teams showed failing and poor measures to follow the protocol, especially in the complete use of protection gear like goggles, facemasks, or surgical caps. The hand hygiene after manipulation of patients was scored low in most of the cases. Both are issues that can be easily improved if an educational process with policies of surveillance of adherence is implemented.

### 4.5. Endotracheal Intubation

ICU patients are dependent on airway devices as part of the respiratory support therapy and to protect airways in neurological injuries. Airway devices like tracheal and tracheostomy tubes are associated with significant risks, especially during initial placement and during subsequent use. In different reviews of unsafe acts, most of the incidents were associated with the initial ETI and postprocedure issues. The most current problems were equipment problems and displacement of tubes, resulting in more than temporary harm to the patients [39–41]. In our study 25% of the procedures were performed with poor measures to follow the protocol and the lowest scored questions were related to the use of appropriate equipment including facemasks and protective lenses. In order to improve adherence, it needs to be an educational process and maintain the general supply of appropriate equipment on daily basis.

### 4.6. Video Observation of the Cases

The method used in this study allows direct observation to objectively evaluate use of protocol measures in contrast with additional ways of reporting adherence. This method allows for better identification of deficiencies especially during emergency procedures and additionally the video can be available for patient safety educational activities. Few studies have used similar methodologies and we encourage patient safety researchers to use it more frequently, especially in areas with a low degree of organization [42, 43]. It would be a useful method for conducting research in other departments such as emergency rooms, operating rooms, or general wards.

### 4.7. Limitations

The study has limitations. It is clear that, in teaching hospitals, members of the team currently in training stages do not perform procedures correctly, and these conditions can influence the results. Additionally we are comparing a general subjective scale used in the hospital patient safety survey instrument with an objective scale to measure patient safety procedures at bedside of the patient. It can introduce a bias but it will also raise a question for the need of specific tools for objective evaluation of patient safety in future studies.

## 5. Conclusion

Perception of patient safety measures was higher than observed in the management of critically ill patients in a teaching hospital in southern Colombia. Infection control was the most frequently underused measure during ICU care. Promotion of ICU bundles of care and frequent audit of their application is a useful option in order to decrease unsafe acts during health care delivery.

## Supplementary Material

The supplementary material includes the patient safety hospital survey and the scale used to evaluate observed adherence.

## Figures and Tables

**Table 1:** Mean level of observed patient safety measures including the 5 different groups of procedures compared with the general perception of patient safety described in the survey.

Level of patient safety measures	Perceived		Observed	
	*n*	%	*n*	%
Excellent	10	27	21	2.5
Very good	14	37.9	73	8.8
Acceptable	9	24.3	79	9.5
Poor	2	5.4	511	61.6
Failing	2	5.4	145	17.6
Total	37	100	829	100

**Table 2:** Level of observed application of patient safety measures recommended for common procedures in the ICU.

Patient safety measures level	PCC protocol *n* (%)	PM protocol *n* (%)	OIP protocol *n* (%)	IC protocol *n* (%)	ETI protocol *n* (%)	Total *n* (%)
Excellent	2 (8.2%)	12 (19.6%)	3 (5.7%)	4 (0.5%)	0 (0%)	21 (2.5%)
Very good	2 (8.2%)	19 (31.1%)	10 (19.2%)	40 (5.8%)	2 (50%)	73 (8.8%)
Acceptable	12 (52.1%)	7 (11.4%)	16 (30.7%)	43 (6.2%)	1 (25%)	79 (9.5%)
Poor	7 (30.4%)	18 (29.5%)	21 (40.3%)	464 (67.3%)	1 (25%)	511 (61.6%)
Failing	0 (0%)	5 (8.1%)	2 (3.8%)	138 (20%)	0 (0%)	145 (17.6%)
Total	**23**	**61**	**52**	**689**	**4**	**829**
